# Giant retroperitoneal lipoma resulting in femoral hernia: A case report

**DOI:** 10.1016/j.ijscr.2025.111584

**Published:** 2025-07-02

**Authors:** Yosuke Kotohata, Akira Umemura, Minoru Sakuraba, Akira Sasaki

**Affiliations:** a Department of Surgery, Iwate Medical University School of Medicine, Shiwa, Japan; b Department of Plastic and Reconstructive Surgery, Iwate Medical University School of Medicine, Shiwa, Japan

**Keywords:** Retroperitoneal lipoma, Femoral hernia, Inguinal hernia, Mesh repair

## Abstract

**Introduction:**

Retroperitoneal lipomas are relatively rare and have nonspecific clinical manifestations. Although only a few cases presenting as inguinal hernias have been reported, no case of herniating through the femoral canal has been reported to our knowledge. Herein we report an extremely rare case of giant retroperitoneal lipoma presented as femoral hernia.

Presentation of case.

A 38-year-old woman was referred to our department with a complaint of right inguinal bulging that began four years earlier and was suspected to be a lipoma or liposarcoma. Although she was asymptomatic and continued following up via computed tomography (CT) scan, she noticed gradual bladder control loss, for which she decided to undergo surgery. During surgery, the retroperitoneal tumor extended to the thigh through the dorsal side of the inguinal ligament. Although we could separate the tumor from the surrounding structures, the defect of the inguinal tissue was extensive because of the tumor's size, and two types of meshes were used to repair the defect. As a result of histopathological examination, the tumor was identified as a lipoma. After the surgery, the patient remains without femoral hernia and no evidence of local recurrence.

**Discussion:**

Retroperitoneal lipomas are rare and can have different clinical manifestations. Surgery is the only treatment for giant lipomas. For extensive tissue defects, a suitable mesh should be selected to prevent postoperative herniation.

**Conclusion:**

Retroperitoneal lipomas sometimes grow to the groin area, and complete resection and repair of the defect are important on surgery.

## Highlights

• Retroperitoneal lipomas resulting in inguinal hernia are extremely rare.
• We experienced a case of giant retroperitoneal lipoma extending into the groin area.
• Surgical resection is only way to treat retroperitoneal lipoma.
• Two layers of mesh may be recommended to repair the large inguinal tissue defect.

## 1 Introduction

The lipoma that occurs in the retroperitoneal space is a rare tumor originating from mesenchymal tissue and requires differentiation from liposarcoma for appropriate treatment strategy. Lipomas mainly occur in the subdermal tissues of the extremities and trunk and rarely in the retroperitoneum.^1^ Some retroperitoneal lipomas extend through the inguinal canal and present as inguinal hernias.[2], [3], [4] Benign tumors sometimes protrude from weak areas of the tissue and cause symptoms. Herein, we report a case of a large retroperitoneal lipoma protruding through the femoral canal, resulting in a femoral hernia. This case report was prepared according to the SCARE guideline.^5^

## 2 Presentation of case

**Fig. 1:**
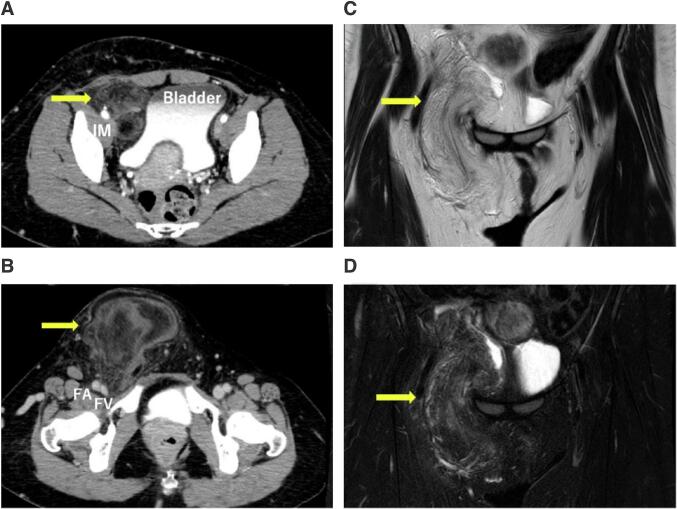
On the CT scan, although the tumor extended from the retroperitoneum into the inguinal region, there was no evidence of bladder invasion (A). In the inguinal region, the tumor was in contact with the femoral artery and vein (B). The tumor had a high signal intensity On T2-weighted MRI imaging (C), and a low signal intensity on fat-suppression MRI imaging (D). IM: iliopsoas muscle, FA: femoral artery, FV: femoral vein.

A 38-year-old woman was referred to our hospital because of a bulge in her right groin that had begun four years earlier. Based on a computed tomography (CT) scan, a lipoma or liposarcoma was suspected. However, considering the asymptomatic nature of the tumor and the risk of intraoperative nerve damage, we continued the follow-up via CT scans. Because she was gradually losing bladder control, we decided to perform complete resection. On admission, her carcinoembryonic antigen (CEA) and carbohydrate antigen 19–9 (CA19–9) levels were unremarkable. The first CT scan revealed a fatty tumor measuring 12 cm in her right groin, which gradually grew to 17 cm in about 5 years. The tumor was lobulated and extended from the retroperitoneal space to the subcutaneous tissue of the groin (Fig. 1A, B). On T1- and T2-weighted magnetic resonance imaging (MRI), the tumor had a high signal intensity similar to that of subcutaneous fat (Fig. 1C). On a fat-suppression MRI, the tumor displayed a low signal intensity (Fig. 1D). We performed an open biopsy to confirm the potential of a malignant tumor, and histopathological examination identified the tumor as a lipoma without malignant potential. However, a pathological diagnosis of the entire tumor was necessary to determine whether it was indeed malignant or not because she was relatively young and suffering from severe symptoms. Based on those results, cancer board meeting was held and we decided to perform complete resection.

**Fig. 2:**
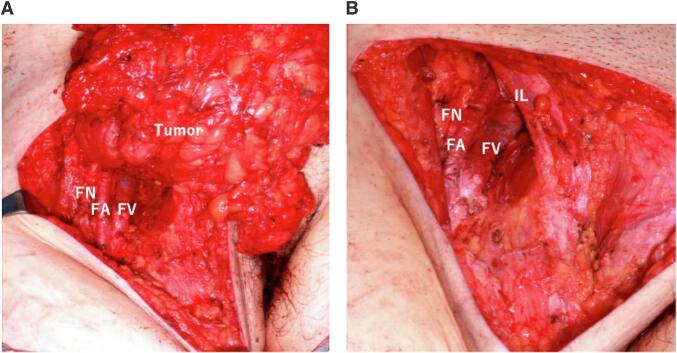
The small vessels supplying the tumor were sectioned, after which we could separate the tumor from the surrounding structures (A). Although the tumor could be removed safely, the inguinal wall defect was large and needed to be repaired(B). FN: femoral nerve, FA: femoral artery, FV: femoral vein, IL: inguinal ligament.

**Fig. 3:**
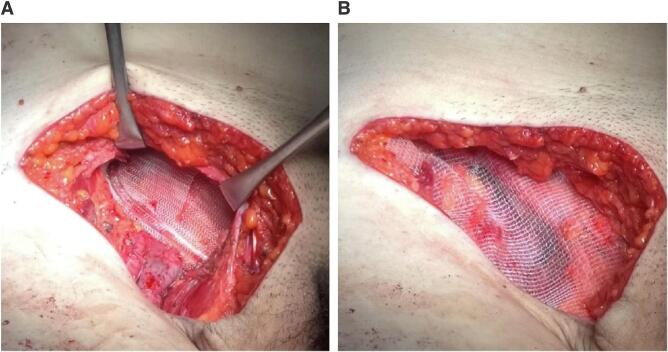
We attached a Kugel patch on the dorsal side of the inguinal ligament (A) and self-grip mesh on its ventral side (B).

The operation was performed under general anesthesia in the supine position, and a 15 cm incision was made in the groin. During surgery, the retroperitoneal tumor extended to the thigh through the dorsal side of the inguinal ligament and was in contact with the femoral artery and vein similar to a femoral hernia (Fig. 2A). However, the mass could be separated from the surrounding structures, which meant we could preserve the blood vessels and femoral nerve (Fig. 2B). The blood vessels flowing into the tumor were ligated and sectioned, and the tumor was completely removed. After the tumor excision, two layers of meshes were used to repair the supporting tissue in the inguinal region. An underlay patch was extended in the preperitoneal space to cover the whole myopectineal orifice in the usual manner (Kugel patch, Bard, U.S.) (Fig. 3A) and a self-grip mesh sized 15 × 5 cm was extended on both the posterior wall of the inguinal canal and the exit of the femoral canal (ProGrip, COVIDIEN, Japan) (Fig. 3B). At the end of the procedure, we sutured the superficial facia and closed the wound by insertion the subcutaneous closed drain to remove exudate and lymph fluid.

**Fig. 4:**
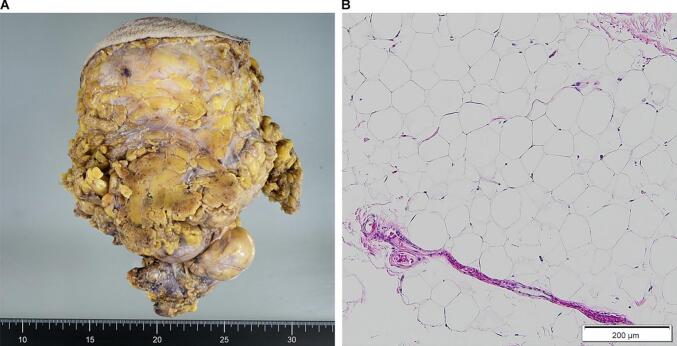
The tumor measured 20 × 17 × 7 cm (A) and had no lipoblasts, atypical spindle cells or other malignant cells. Histopathological examination showed relatively uniform mature adipocytes forming lobular structures while being densely constructed (B).

The tumor measured 20 × 17 × 7 cm and weighted 1750 g (Fig. 4A). A postoperative histopathological examination showed relatively uniform mature adipocytes forming lobular structures while being densely constructed (Fig. 4B). Immunostaining showed that CDK4 and MDM2 were negative. No lipoblasts, atypical spindle cells or other malignant cells were found; therefore, the tumor was identified as a lipoma.

Postoperatively, the patient had no bleeding, infection or neurological symptoms. Although the length of her hospital stay was prolonged due to lymphorrhea, the amount of drain fluid gradually decreased, and the drain was removed on postoperative day 14. The patient was discharged from the hospital on day 15. After surgery, bladder control loss improved and the patient showed no sign of a femoral hernia. At 6-month after surgery follow-up, there was no recurrence of the retroperitoneal lipoma.

## 3 Discussion

Retroperitoneal lipomas are relatively rare, and only 17 cases were reported between 1980 and 2013.^6^ In. addition, lipomas that herniate into the inguinal region are less reported. Lipomas account for only 2.9 % of all primary retroperitoneal tumors, and about 70 % of primary retroperitoneal tumors are malignant neoplasms.^7^ The clinical manifestations of retroperitoneal lipomas are nonspecific, although they sometimes present with abdominal swelling, digestive symptoms, and signs of urinary tract compression.^2^^,^[8], [9], [10], [11] Conversely, weight loss and abdominal enlargement are the most common clinical manifestations of malignant neoplasms of the retroperitoneum.^12^ In this case, the loss of bladder control mainly occurred due to bladder compression caused by enlargement of the tumor.

Although adequate preoperative differentiation between high-grade liposarcoma and lipoma is important in choosing the appropriate treatment, it is difficult to distinguish these conditions with CT or MRI because the image findings are very similar. In this case, the tumor was diagnosed as a lipoma by an open biopsy before complete resection, and the onset and progression of symptoms were the conclusive factor for surgery. In addition, if malignancy cannot be ruled out, complete resection is the ultimate treatment. Resection is the first-choice treatment for retroperitoneal lipomas. However, in this case, a comprehensive judgment was required based on the clinical presentation, the patient's wishes, and the risk of intraoperative damage to the surrounding organs. Importantly, to achieve a complete resection, a sound plan was needed before surgery. Regarding the extensive postresection tissue defect, we preoperatively discussed the necessity and method of defect repair. If vascular invasion is suspected in such cases, combined resection and vessel anastomosis should be considered.

To the best of our knowledge, only three cases of retroperitoneal lipoma in which the tumor herniated to the inguinal region have been reported thus far (Table 1). In previous cases, the tumors had spread into the inguinal canal, whereas in our case, the tumor had spread through the femoral canal. In the previous cases, underlay mesh was employed, or a simple inguinal canal closure was performed to prevent hernia recurrence. In one case, it was not revealed how they repaired the defect. In this case, the defect of the inguinal tissue due to tumor resection was large, rendering simple closure impossible. In addition, it was thought that a repair performed using only an underlay mesh could lead to a femoral hernia through the gap in the damaged inguinal ligament. Therefore, we also attached self-grip mesh as an onlay mesh to the ventral side of the inguinal ligament, and as a result, we used two layers of mesh to prevent femoral hernia, as in the Lichtenstein procedure. There are several hernia repair techniques, and the appropriate choice depends on the size and location of the defect. In addition, it is important to monitor patient's symptoms after surgery to check for hernia development.

**Table 1:** Reported cases of retroperitoneal lipomas herniating into the inguinal region. NA: Not available.

	Year	Age	Sex	Passing canal	Size/Weight	Repair
Singh et al.^2^	2011	65	Male	Inguinal canal	25 × 12 cm/NA	Written as only simple closure of the inguinal canal
Nardi et al.^3^	2021	34	Female	Inguinal canal	24 × 13 × 7 cm/636 g	NA
Sabino et al.^4^	2023	30	Female	Inguinal canal	20 × 11 × 11 cm/1000 g	Polypropylene mesh (15 × 15 cm)
Our case	2025	38	Female	Femoral ring	20 × 17 × 7 cm/1750 g	Kugel patch and self-grip mesh

## 4 Conclusion

We report a case of a giant retroperitoneal lipoma resulting in a femoral hernia. There were no previous reports of a retroperitoneal lipoma extending to the thigh through the femoral canal. To resect completely, it is essential to prepare sufficient surgical plans and consider appropriate repair techniques. If the defect is large, because the underlay mesh can be unfastened, we think that using an onlay mesh in combination may be recommended.

## Consent

Written informed consent was obtained from the patient for the publication of this case report and the accompanying images. A copy of the consent form is available for review by the target journal's Editor-in-Chief of this journal upon request.

## Ethical approval

The requirement for ethical approval was waived by the Clinical Research Ethics.

## Funding

We declare that no funding was receiver in support of this work.

## Author contribution

Yosuke Kotohata and Akira Umemura conceived the case presentation and drafted the manuscript.

Akira Umemura, Minoru Sakuraba and Akira Sasaki read and approved the final manuscript.

## Guarantor

Akira Umemura.

## Declaration of competing interest

None.
